# Cytokine and Chemokine Secretome and Risk of CMV Infection Following Discontinuation of Valganciclovir Prophylaxis

**DOI:** 10.3389/ti.2023.10979

**Published:** 2023-01-26

**Authors:** Mario Fernández-Ruiz, Patricia Parra, Tamara Ruiz-Merlo, Natalia Redondo, Isabel Rodríguez-Goncer, Amado Andrés, José María Aguado

**Affiliations:** ^1^ Unit of Infectious Diseases, Hospital Universitario “12 de Octubre”, Instituto de Investigación Sanitaria Hospital “12 de Octubre” (imas12), Madrid, Spain; ^2^ Centro de Investigación Biomédica en Red de Enfermedades Infecciosas (CIBERINFEC), Instituto de Salud Carlos III (ISCIII), Madrid, Spain; ^3^ Department of Medicine, School of Medicine, Universidad Complutense, Madrid, Spain; ^4^ Department of Nephrology, Hospital Universitario “12 de Octubre”, Instituto de Investigación Sanitaria Hospital “12 de Octubre” (imas12), Madrid, Spain

**Keywords:** kidney transplantation, cytomegalovirus, cytokine, chemokine, IP-10

Dear Editors,

The advent of interferon (IFN)-γ release assays (IGRAs) to quantify cytomegalovirus (CMV)-specific cell-mediated immunity (CMV-IMC) has represented a major step in the effort to individualize preventive strategies after kidney transplantation (KT). We have recently shown, however, that the QuantiFERON®-CMV (QTF-CMV) assay at the time of discontinuation of antiviral prophylaxis exhibits suboptimal accuracy (sensitivity of 77.4%, specificity of 34.3%, positive [PPV] and negative predictive values [NPV] of 64.1% and 50.0%, respectively) to predict protection among KT recipients that had received induction therapy with antithymocyte globulin (ATG) (1). The assessment of IFN-γ production by IGRAs is aimed at recapitulating the Th1-polarized CMV-IMC. Nevertheless, CD4^+^ T-cell functions are also mediated through other lymphocyte subsets (such as Th2 or Th17), each of which secretes a distinct cytokine profile. A comprehensive profiling of the cytokine and chemokine responses upon CMV-specific stimulation may improve the performance of the QTF-CMV assay (2).

Samples collected from patients recruited in a previous study were used for the present analysis (1). Consecutive CMV-seropositive KT recipients receiving ATG induction at our institution between April 2015 and June 2018 underwent CMV-CMI monitoring by the QTF-CMV assay at months 2, 3, 4 and 5. A 3-month course of valganciclovir prophylaxis was scheduled in all of them. The QTF-CMV assay was performed according to the manufacturer’s instructions. We selected those samples obtained at the time of discontinuation of prophylaxis (±3 weeks). The following 27 cytokines and chemokines were measured in stimulated (“CMV tube”) and unstimulated (“nil tube”) plasma samples by means of analyte-specific capture beads coated with target-specific capture antibodies in a Luminex® 200 instrument: IFN-γ, interleukin [IL]-1β, IL-2, IL-4, IL-5, IL-6, IL-8, IL-9, IL-10, IL-12p70, IL-13, IL-17A, IL-18, IL-21, IL-22, IL-23, IL-27, tumor necrosis factor [TNF]-α, eotaxin (CCL11), GRO α (CXCL1), IP-10 (CXCL10), SDF-1α (CXCL12), MCP-1 (CCL2), MCP-2 (CCL8), MIP-1α (CCL3), MIP-1β (CCL4) and RANTES (CCL5) (Th1/Th2 Cytokine & Chemokine 20-Plex ProcartaPlex™ Panel 1, Th9/Th17/Th22 Cytokine 7-Plex ProcartaPlex™ Panel, and MCP-2 ProcartaPlex™ Simplex kits, all from Thermo Fisher Scientific, Waltham, MA). The study outcome was the occurrence of clinically significant (DNAemia >1,000 IU/mL) CMV infection (asymptomatic viremia or clinical disease) from the discontinuation of prophylaxis to post-transplant month 12. Further details are provided in Supplementary Methods.

We included 78 KT recipients (Supplementary Table S1, Supplementary Results), 13 of which developed clinically significant CMV infection following discontinuation of valganciclovir prophylaxis (12-month incidence: 17.9%). The median interval between the timing of sampling and the end of prophylaxis, on one hand, and the occurrence of CMV infection, on the other hand, were 35.0 (interquartile range [IQR]: 24.0–70.0) and 45.0 (IQR: 33.0–56.0) days, respectively. The analysis of the blood secretome after CMV-specific stimulation revealed detectable levels in the majority of patients, since only 5 cytokines (IL-5, IL-9, IL-17A, IL-22, IL-23 and IL-27) were detected in less than 50% of specimens. The comparison of cytokine/chemokine expression at baseline and following stimulation (“CMV tube” minus “nil tube”) showed a significant increase (Δ >0) of IFN-γ, IL-18, IP-10 and MCP-2, whereas IL-1β, IL-6, IL-10, TNF-α and MIP-1α were downregulated (Δ ≤0) (Supplementary Table S2). The heatmap of cytokines/chemokines correlations—once unstimulated samples were subtracted from the CMV peptide-stimulated samples—is shown in Figure 1A. The highest correlations were found between IFN-γ and MCP-2 and IP-10 levels (Spearman’s rho coefficients = 0.766 and 0.726, respectively; *p*-values < 0.00001).

**FIGURE 1:**
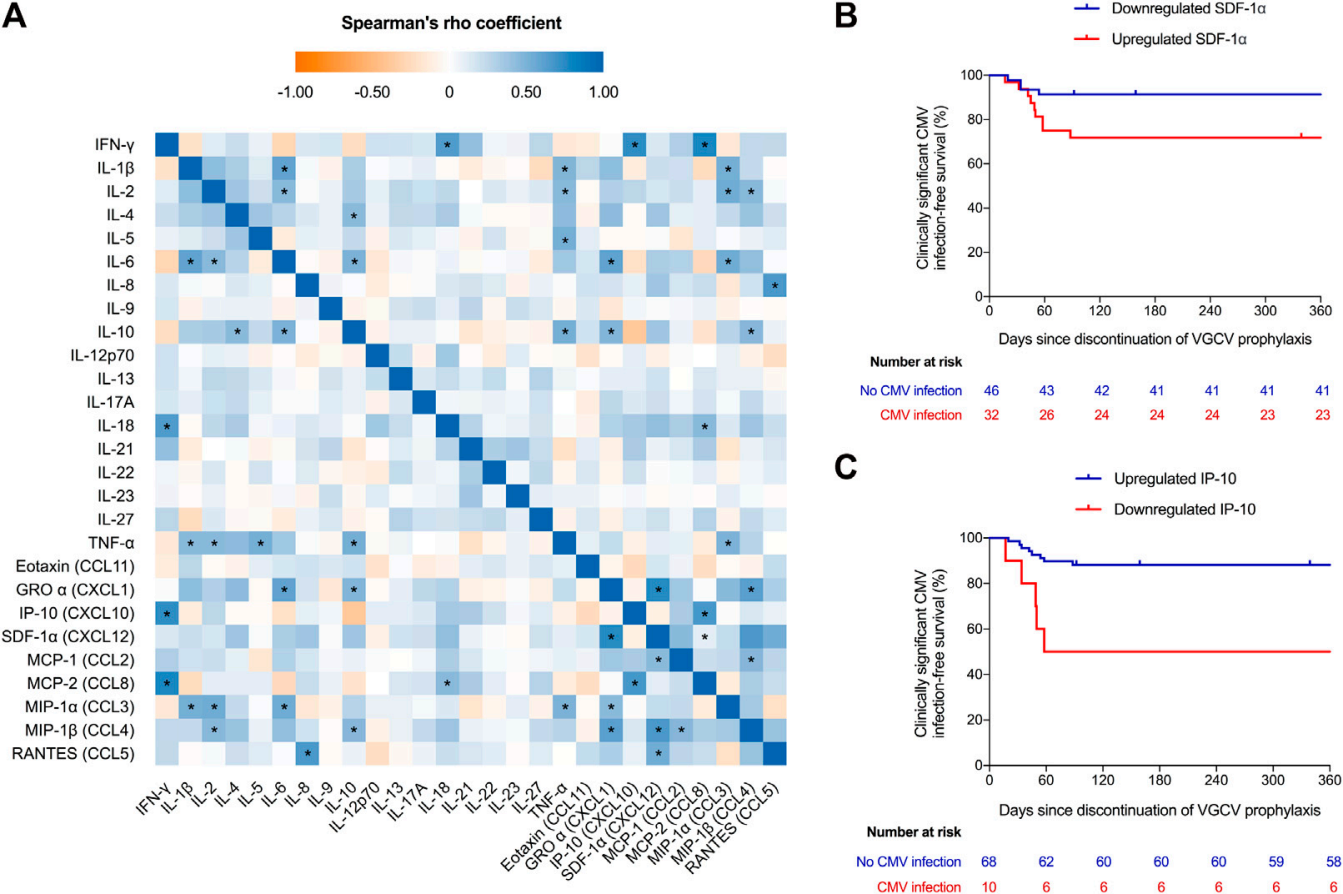
**(A)** Heatmap of the correlation between analyzed cytokines and chemokines. Values obtained in unstimulated plasma samples were subtracted from CMV peptide-stimulated samples (“CMV tube” minus “nil tube”). **(B)** Clinically significant (DNAemia >1,000 IU/mL) CMV infection-free survival after the discontinuation of valganciclovir prophylaxis according to expression (upregulation or downregulation) of IP-10 and **(C)** SDF-1α upon whole blood stimulation with CMV-specific viral peptides (log-rank *p*-values = 0.001 and 0.027, respectively). Three patients were censored at days 92, 159 and 339 because of graft loss (rejection), loss to follow-up and death (gastric adenocarcinoma), respectively. The criteria for classifying a given recipient in the upregulated or regulated categories was that the increment (Δ) in plasma IP-10 or SDF-1α levels from unstimulated baseline samples to CMV peptide-stimulated samples (“CMV tube” minus “nil tube”) was positive (Δ >0) or zero or negative (Δ ≤0), respectively. * *p-*value < 0.00007 (Bonferroni-corrected threshold for statistical significance). CMV, cytomegalovirus; GRO α, growth-related onconge α; IFN, interferon; IL, interleukin; IP-10, interferon-γ-inducible protein 10; MCP, monocyte chemoattractant protein; MIP-1, macrophage inflammatory protein 1; RANTES, regulated upon activation, normal T-cell expressed and presumably secreted; SDF-1α, stromal cell-derived factor 1α; TNF, tumor necrosis factor-α; VGCV, valganciclovir.

We analyzed the blood secretome according to the occurrence of clinically significant CMV infection after cessation of prophylaxis. Upregulation of IP-10 and IL-2 and downregulation of SDF-1α were significantly associated with a lower risk of this outcome, with the strongest associations observed for IP-10 and SDF-1α (Supplementary Table S3). The 12-month clinically significant CMV infection-free survival was higher in recipients in which IP-10 levels increased (hazard ratio [HR]: 0.188; 95% confidence interval [CI]: 0.061–0.577; *p*-value = 0.003) (Figure 1B) and SDF-1α levels decreased (HR: 0.288; 95% CI: 0.09–0.937; *p*-value = 0.039) following stimulation (Figure 1C). Older patient age (54.6 ± 11.5 versus 43.9 ± 9.1 years; *p*-value = 0.006) (Supplementary Table S4) and lower number of ATG doses (median: 4.7 [IQR: 1.5–6.6] versus 5.6 [IQR: 1.7–6.9] doses; *p*-value = 0.016) (Supplementary Table S5) were associated with IP-10 upregulation and SDF-1α downregulation, respectively. By applying IP-10 upregulation as diagnostic criteria, the sensitivity, specificity, PPV and NPV to predict effective protection from clinically significant CMV infection were 92.3%, 38.5%, 88.2% and 50.0%, respectively.

We have characterized the cytokine and chemokine secretome in whole blood samples from seropositive KT recipients stimulated with a pool of CMV peptides contained in the commercial QTF-CMV assay. Not surprisingly, IL-18, IP-10 and MCP-2 levels were found to be upregulated and strongly correlate with IFN-γ. Indeed, IL-18 is a potent inducer of IFN-γ production (3), whereas IP-10 expression is activated by the IFN-γ-signaling in several cell types. Of note, IP-10 circulates at much higher levels than IFN-γ and plays a role in the generation and function of effector T-cells (4). Therefore, it has been proposed that the detection of IP-10 may serve as a convenient alternative to IGRA for the diagnosis of latent tuberculosis infection (5, 6).

In our experience, the demonstration of IP-10 upregulation in response to CMV peptides yielded a better diagnostic accuracy in terms of sensitivity and PPV to predict immune protection than the cut-off for IFN-γ proposed by the manufacturer (≥0.2 IU/mL) in the QTF-CMV assay (1). Since specificity and NPV improved only marginally, it is likely that pathways different from the IFN-γ/IP-10 axis may be involved in conferring protection against CMV. In addition, a relatively high proportion of patients were apparently protected (only 17.9% developed the outcome), which lowers the NPV. By using a similar approach, Lisboa et al. also found that MCP-2 and IP-10 were the cytokines/chemokines showing the highest expression increase upon CMV peptide stimulation, with close correlation with IFN-γ production. These authors reported an excellent discriminatory capacity to predict spontaneous CMV viremia clearance for both chemokines (2). Differences in analyzed outcomes and CMV serostatus may explain the discordance regarding the predictive role of MCP-2 between the study by Lisboa et al. (2) and ours.

In addition, we report the novel observation that SDF-1α downregulation is predictive of protection against CMV. Often considered a homeostatic chemokine, inflammatory activities have been attributed to SDF-1 (CXCL12) (7). Polymorphisms in the *CXCL12* gene are associated with the occurrence of CMV reactivation after allogeneic hematopoietic stem cell transplantation (8), whereas elevated plasma levels identify poor immune reconstitution in HIV patients (9). SDF1 mainly signals *via* the CXCR4 receptor, and it has been shown that CMV enhances SDF-1/CXCR4 signaling during infection through the product of the *UL111A* gene (which encodes a viral ortholog of human IL-10) (10). It may be hypothesized that the downregulation of SDF-1α expression would partially abrogate this immune evasion mechanism, leading to a decreased host susceptibility to CMV replication.

Due to the small sample size of our study, the present results should be confirmed in a larger prospective cohort, as well as its potential application to the clinical decision-making process.

## Data Availability

The raw data supporting the conclusion of this article will be made available by the authors, without undue reservation.
