# Supplementary material for: Cytokine and Chemokine Secretome and Risk of CMV Infection Following Discontinuation of Valganciclovir Prophylaxis
Source: Transpl Int. 2023 Jan 26;36:10979. doi: 10.3389/ti.2023.10979 (PMC9908579; doi:10.3389/ti.2023.10979)
Supplement: Supplementary file 1 [file DataSheet1.PDF]

Supporting Material

Supplementary Methods

QuantiFERON-CMV assay

The QuantiFERON-CMV assay (Qiagen GmbH, Hilden, Germany) was performed according to manufacturer's instructions. One-mL whole blood aliquots were immediately transferred into three tubes: the "cytomegalovirus (CMV) tube" contained a pool of 22 viral peptides mapped within pp65, pp50, immediate-early (IE)-1, IE-2 and glycoprotein B antigens and restricted by widespread human leukocyte antigen (HLA) class I molecules; the "mitogen tube" contained a lymphocyte mitogen (phytohemagglutinin) as positive control; and the "nil tube" contained heparin (negative control). These tubes were shaken vigorously for 5 seconds and incubated overnight at 37°C. Plasma supernatants were harvested and frozen at -80°C until analysis.

Statistical analysis

The Wilcoxon matched-pairs signed-rank test was used to compare cytokine/chemokine levels between unstimulated and CMV peptide-stimulated samples. Correlations were explored with the Spearman's rank correlation coefficient. Blood secretome in kidney transplant recipients that developed or not the outcome was compared using the χ^2 test. The criteria used for classifying a patient in the upregulated or regulated categories for a given cytokine/chemokine was that the increment (Δ) in plasma levels for that biomarker from unstimulated baseline samples to CMV peptide-stimulated samples (i.e. "CMV tube" minus "nil tube") was positive (Δ >0) or zero or negative (Δ ≤0), respectively. The Bonferroni correction was applied to account for potential *P*-value inflation. In addition to the study outcome of clinically significant CMV infection (DNAemia >1,000 IU/mL), the clinical factors associated with change patterns (upregulation or downregulation) in cytokine/chemokine secretome that exhibited a protective role against CMV were also explored. To this end, the χ^2 test or Fisher's exact test were used to compare categorical variables, whereas the Student's t-test or Mann–Whitney U test were applied for continuous variables. A *P*-value <0.05 was considered significant. SPSS version 22.0 (IBM Corporation®, Armonk, NY) was used for statistical analysis. Statistical analysis was performed with SPSS version 20.0 (IBM Corp., Armonk, NY).

Supplementary Results

Table S1. Clinical features and post-transplant events in the study population (n = 78).

Variable	
Age of recipient, years [mean ± SD]	53.2 ± 11.7
Male gender [n (%)]	47 (60.3)
BMI at transplantation, Kg/m^2 [mean ± SD]	25.5 ± 6.1
Pre-transplant comorbidities [n (%)]	
Hypertension	64 (82.1)
Diabetes mellitus	20 (25.6)
Atherotrombotic disease[a]	16 (20.5)
Coronary heart disease	7 (9.0)
Non-ischemic heart disease	13 (16.7)
Chronic pulmonary disease	8 (10.3)
Cerebrovascular disease	7 (9.0)
Previous kidney transplantation [n (%)]	23 (29.5)
Underlying end-stage renal disease [n (%)]	
Diabetic nephropathy	15 (19.2)
Polycystic kidney disease	13 (16.7)
Glomerulonephritis	6 (7.7)
IgA nephropathy	5 (6.4)
Reflux nephropathy	5 (6.4)
Nephroangiosclerosis	4 (5.1)
Chronic interstitial nephropathy	2 (2.6)
Lupus nephropathy	2 (2.6)
Congenital nephropathy	2 (2.6)
Unknown	11 (14.1)
Other	13 (16.7)
CMV serostatus [n (%)]	
D+/R+	64 (82.1)
D-/R+	13 (16.7)
D unknown/R+	1 (1.3)
Positive EBV serostatus (anti-EBNA IgG) [n (%)]	74 (94.9)
Positive HCV serostatus [n (%)]	14 (17.9)
Pre-transplant renal replacement therapy [n (%)]	75 (96.2)
Time on dialysis, months [median (IQR)]	33.4 (17.2 – 63.4)
Type of transplantation [n (%)]	
Single kidney	75 (96.2)
Simultaneous pancreas-kidney	3 (3.8)
Type of donor [n (%)]	
DBD donor	43 (55.1)
DCD donor	34 (43.6)
Living donor	1 (1.3)
Cold ischemia time, hours [mean ± SD]	17.9 ± 6.5
Number of HLA mismatches [median (IQR)]	4 (3 – 5)

Total number of ATG doses [median (IQR)]	5 (3 – 6.3)
Primary immunosuppression regimen [n (%)]	
Prednisone, tacrolimus, MMF/MPA	73 (93.6)
Prednisone, tacrolimus, azathioprine	5 (6.4)
Duration of VGCV prophylaxis, days [median (IQR)]	91 (90 – 96.8)
CMV events by post-transplant month 12 [n (%)]	
Any CMV infection	30 (38.5)
Time from transplantation, days [median (IQR)][b]	150 (126.5 – 183.5)
Any CMV infection after discontinuation of VGCV prophylaxis	29 (37.2)
Time from discontinuation, days [median (IQR)][b]	54 (42 – 84)
Clinically significant CMV infection after discontinuation of VGCV prophylaxis	14 (17.9)
Time from discontinuation, days [median (IQR)][b]	47 (33.5 – 58)
CMV disease after discontinuation of VGCV prophylaxis	3 (3.8)
Other complications by post-transplant month 12 [n (%)]	
Delayed graft function[c]	44 (56.4)
Number of dialysis sessions [median (IQR)]	2.5 (1 – 4)
Development of NODAT	15 (19.2)
Development of *de novo* DSA	7 (9.0)
Renal artery stenosis requiring revascularization	6 (7.7)
Biopsy-proven acute graft rejection	7 (9.0)
Time from transplantation, days [median (IQR)][b]	44 (19 – 146)

ATG: antithymocyte globulin; BMI: body mass index; CMV: cytomegalovirus; D: donor; DBD: donation after brain death; DCD: donation after circulatory death; DSA: donor-specific antibody; EBV: Epstein-Barr virus; EBNA: EBV nuclear antigen; HLA: human leukocyte antigen; HCV: hepatitis C virus; IQR: interquartile range; MPA/MMF: mycophenolic acid / mycophenolate mofetil; NODAT: new-onset diabetes mellitus after transplantation; R: recipient; SD: standard deviation; VGCV: valganciclovir.

[a] Includes coronary artery disease, carotid artery stenosis, abdominal aortic aneurysm, and/or peripheral arterial disease of lower limbs.

[b] Time interval to the first episode.

[c] Defined by the requirement of hemodialysis within the first two post-transplant weeks.

Table S2. Description of cytokine and chemokine secretome upon whole blood stimulation with a pool of CMV-specific viral peptides mapped within pp65, pp50, IE-1, IE-2 and glycoprotein B antigens and restricted by HLA class I molecules.

**Cytokine / chemokine**	**Unstimulated samples ("nil tube")**	**CMV peptide-stimulated samples ("CMV tube")**		**Effect of peptide stimulation ("CMV tube" minus "nil tube")**	
	**Plasma levels, pg/mL** [median (IQR)]	**Samples with detectable analyte** [n (%)][a]	**Plasma levels, pg/mL** [median (IQR)]	**Behaviour**	***P*-value**[b]
IFN-γ	26.4 (17.2 – 40.9)	78 (100.0)	48.3 (23.9 – 79.8)	**Upregulated**	**<0.0001**
IL-1β	374.6 (170.3 – 653.3)	78 (100.0)	275.7 (114.1 – 437.8)	**Downregulated**	**<0.001**
IL-2	15.9 (10.3 – 24.4)	73 (93.6)	15.9 (9.4 – 25.4)	Downregulated	0.336
IL-4	12.2 (8.8 – 21.5)	73 (93.6)	11.7 (8.6 – 17.8)	Downregulated	0.072
IL-5	0.0 (0.0 – 0.2)	13 (16.7)	0.0 (0.0 – 0.0)	Downregulated	0.004
IL-6	2,159.0 (938.8 – 4,540.9)	78 (100.0)	1,412.9 (798.2 – 2,970.6)	**Downregulated**	**<0.0001**
IL-8	3,719.8 (819.4 – 7,690.9)	78 (100.0)	4,048.2 /845.1 – 9,352.4)	Upregulated	0.079
IL-9	0.0 (0.0 – 0.0)	10 (12.8)	0.0 (0.0 – 0.0)	Downregulated	0.533
IL-10	7.3 (4.8 – 17.8)	78 (100.0)	3.9 (2.1 – 8.3)	**Downregulated**	**<0.0001**
IL-12p70	3.3 (0.0 – 5.8)	56 (71.8)	3.3 (0 – 6.3)	Upregulated	0.697
IL-13	2.1 (1.4 – 2.7)	61 (78.2)	2.1 (1.4 – 2.8)	Upregulated	0.681
IL-17A	0.0 (0.0 – 0.0)	11 (14.1)	0.0 (0.0 – 0.0)	Upregulated	0.022
IL-18	46.2 (25.9 – 81.9)	78 (100.0)	57.7 (29.1 – 104.7)	**Upregulated**	**<0.0001**
IL-21	3.2 (0.9 – 4.6)	59 (75.6)	3.1 (0.3 – 5.5)	Downregulated	0.344
IL-22	0.0 (0.0 – 0.5)	16 (20.5)	0.0 (0.0 – 0.0)	Downregulated	0.422

IL-23	0.0 (0.0 – 4.1)	23 (29.5)	0.0 (0.0 – 3.9)	Upregulated	0.980
IL-27	0.0 (0.0 – 13.6)	35 (44.9)	0.0 (0.0 – 16.5)	Upregulated	0.396
TNF-α	106.0 (48.1 – 254.7)	78 (100.0)	79.9 (42.7 – 193.9)	**Downregulated**	**<0.001**
Eotaxin (CCL11)	129.9 (85.8 – 227.9)	78 (100.0)	123.8 (77.9 – 207.4)	Downregulated	0.018
GRO α (CXCL1)	194.8 (103.1 – 520.4)	78 (100.0)	157.9 (86.4 – 314.9)	Downregulated	0.007
IP-10 (CXCL10)	62.3 (40.9 – 105.7)	78 (100.0)	350.6 (124.5 – 1,192.5)	**Upregulated**	**<0.0001**
SDF-1α (CXCL12)	1,588.2 (1,005.5 – 2,110.6)	78 (100.0)	1,455.2 (1,150.0 – 2,077.1)	Downregulated	0.279
MCP-1 (CCL2)	1,538.6 – 3,302.2)	78 (100.0)	2,542.9 (976.4 – 3,659.0)	Upregulated	0.038
MCP-2 (CCL8)	23.4 (15.7 – 38.3)	78 (100.0)	118.9 (41.0 – 287.9)	**Upregulated**	**<0.0001**
MIP-1α (CCL3)	689.4 (424.3 – 1,319.0)	78 (100.0)	562.3 (339.3 – 1,112.3)	**Downregulated**	**<0.0001**
MIP-1β (CCL4)	2,774.5 (792.7 – 4,857.6)	78 (100.0)	2,961.5 (755.1 – 6,335.8)	Upregulated	0.191
RANTES (CCL5)	150.5 (93.6 – 224.5)	78 (100.0)	172.6 (100.1 – 253.5)	Upregulated	0.002

GRO α: growth-related onconge α; IQR: interquartile range; IL: interleukin; IFN: interferon; IP-10: interferon-γ-inducible protein 10; MCP: monocyte chemoattractant protein; MIP-1: macrophage inflammatory protein 1; RANTES: regulated upon activation, normal T-cell expressed and presumably secreted; SDF-1α: stromal cell-derived factor 1α; TNF: tumor necrosis factor-α.

[a] Above the lower limit of detection for the corresponding cytokine or chemokine.

[b] Bonferroni-corrected *P*-value threshold for statistical significance = 0.0018. Bold characters denote significant differences.

Table S3. Occurrence of clinically significant (DNAemia >1,000 IU/mL) CMV infection after discontinuation of valganciclovir prophylaxis according to the cytokine and chemokine secretome upon whole blood stimulation with CMV-specific viral peptides.

**Cytokine / chemokine**	**Behaviour**	**Clinically significant CMV infection** (n = 13)	**No clinically significant CMV infection** (n = 65)	***P*-value**
IFN-γ	Upregulated	11 (84.6)	55 (84.6)	1.000
	Downregulated	2 (15.4)	10 (15.4)	
IL-1β	Upregulated	4 (30.8)	20 (30.8)	1.000
	Downregulated	9 (69.2)	45 (69.2)	
IL-2	Upregulated	2 (15.4)	29 (44.6)	**0.049**
	Downregulated	11 (84.6)	36 (55.4)	
IL-4	Upregulated	6 (46.2)	21 (32.3)	0.355
	Downregulated	7 (53.8)	44 (67.7)	
IL-5	Upregulated	0 (0.0)	4 (6.2)	1.000
	Downregulated	13 (100.0)	61 (93.8)	
IL-6	Upregulated	1 (7.7)	19 (29.2)	0.165
	Downregulated	12 (92.3)	46 (70.8)	
IL-8	Upregulated	9 (69.2)	37 (56.9)	0.410
	Downregulated	4 (30.8)	28 (43.1)	
IL-9	Upregulated	0 (0.0)	4 (6.2)	1.000
	Downregulated	13 (100.0)	61 (93.8)	
IL-10	Upregulated	4 (30.8)	11 (16.9)	0.262
	Downregulated	9 (69.2)	54 (83.1)	
IL-12p70	Upregulated	6 (46.2)	17 (26.2)	0.187
	Downregulated	7 (53.8)	48 (73.8)	
IL-13	Upregulated	4 (30.8)	19 (29.2)	1.000
	Downregulated	9 (69.2)	46 (70.8)	
IL-17A	Upregulated	2 (15.4)	9 (13.8)	1.000
	Downregulated	11 (84.6)	56 (86.2)	
IL-18	Upregulated	10 (76.9)	48 (73.8)	1.000
	Downregulated	3 (23.1)	17 (26.2)	
IL-21	Upregulated	3 (23.1)	21 (32.3)	0.744
	Downregulated	10 (76.9)	44 (67.7)	
IL-22	Upregulated	0 (0.0)	9 (13.8)	0.342
	Downregulated	13 (100.0)	56 (86.2)	
IL-23	Upregulated	2 (15.4)	11 (16.9)	1.000

	Downregulated	11 (84.6)	54 (83.1)	
IL-27	Upregulated	2 (15.4)	16 (24.6)	0.721
	Downregulated	11 (84.6)	49 (75.4)	
TNF-α	Upregulated	4 (30.8)	17 (26.2)	0.740
	Downregulated	9 (69.2)	48 (73.8)	
Eotaxin (CCL11)	Upregulated	7 (53.8)	27 (41.5)	0.414
	Downregulated	6 (46.2)	38 (58.5)	
GRO α (CXCL1)	Upregulated	6 (46.2)	23 (35.4)	0.536
	Downregulated	7 (53.8)	42 (64.6)	
IP-10 (CXCL10)	Upregulated	8 (61.5)	60 (92.3)	**0.009**
	Downregulated	5 (38.5)	5 (7.7)	
SDF-1α (CXCL12)	Upregulated	9 (69.2)	23 (35.4)	**0.024**
	Downregulated	4 (30.8)	42 (64.6)	
MCP-1 (CCL2)	Upregulated	8 (61.5)	41 (63.1)	1.000
	Downregulated	5 (38.5)	24 (36.9)	
MCP-2 (CCL8)	Upregulated	10 (76.9)	62 (95.4)	0.055
	Downregulated	3 (23.1)	3 (4.6)	
MIP-1α (CCL3)	Upregulated	1 (7.7)	16 (24.6)	0.277
	Downregulated	12 (92.3)	49 (75.4)	
MIP-1β (CCL4)	Upregulated	7 (53.8)	39 (60.0)	0.680
	Downregulated	6 (46.2)	26 (40.0)	
RANTES (CCL5)	Upregulated	7 (53.8)	44 (67.7)	0.355
	Downregulated	6 (46.2)	21 (32.3)	

CMV: cytomegalovirus; GRO α: growth-related onconge α; IQR: interquartile range; IL: interleukin; IFN: interferon; IP-10: interferon-γ-inducible protein 10; MCP: monocyte chemoattractant protein; MIP-1: macrophage inflammatory protein 1; RANTES: regulated upon activation, normal T-cell expressed and presumably secreted; SDF-1α: stromal cell-derived factor 1α; TNF: tumor necrosis factor-α.

Table S4. Clinical factors associated with IP-10 upregulation upon whole blood stimulation with CMV-specific viral peptides (n = 78).

**Variable**	**IP-10 upregulation** (n = 68)	**No IP-10 upregulation** (n = 10)	***P*-value**[a]
Age of recipient, years [mean ± SD]	54.6 ± 11.5	43.9 ± 9.1	**0.006**
Male gender [n (%)]	41 (60.3)	6 (60.0)	1.000
BMI at transplantation, Kg/m^2 [mean ± SD]	25.2 ± 4.4	28.0 ± 13.8	0.580
Pre-transplant comorbidities [n (%)]			
Hypertension	57 (83.8)	7 (70.0)	0.373
Diabetes mellitus	18 (26.5)	2 (20.0)	1.000
Atherothrombotic disease[b]	16 (23.5)	0 (0.0)	0.112
Coronary heart disease	7 (10.3)	0 (0.0)	0.586
Non-ischemic heart disease	13 (19.1)	0 (0.0)	0.130
Chronic pulmonary disease	6 (8.8)	2 (20.0)	0.271
Cerebrovascular disease	7 (10.3)	0 (0.0)	0.568
Previous kidney transplantation [n (%)]	17 (25.4)	6 (60.0)	0.057
Underlying end-stage renal disease [n (%)]			
Diabetic nephropathy	14 (20.6)	1 (10.0)	0.427
Polycystic kidney disease	10 (14.7)	3 (30.0)	0.226
Glomerulonephritis	5 (7.4)	1 (0.0)	0.769
IgA nephropathy	5 (7.4)	0 (0.0)	1.000
Reflux nephropathy	3 (4.4)	2 (20.0)	0.120
D-/R+ CMV serostatus [n (%)]	11 (16.2)	2 (20.0)	0.670
Positive EBV serostatus (anti-EBNA IgG) [n (%)]	65 (95.6)	9 (90.0)	0.454
Positive HCV serostatus [n (%)]	14 (20.6)	0 (0.0)	0.194
Pre-transplant renal replacement therapy [n (%)]	65 (95.6)	10 (100.0)	1.000
Time on dialysis, months [median (IQR)]	24.5 (14.8 – 60.2)	47.8 (26.7 – 104.5)	0.175

Single kidney transplantation [n (%)]	66 (97.1)	9 (90.0)	0.341
DCD donor [n (%)]	31 (45.6)	3 (30.0)	0.500
Cold ischemia time, hours [mean ± SD]	17.8 ± 6.7	18.2 ± 5.0	0.861
Number of HLA mismatches [median (IQR)]	4 (3 – 5)	4 (3 – 5.3)	0.908
Total number of ATG doses [median (IQR)]	4.9 (1.7 – 6.9)	3.9 (1.3 – 6.9)	0.670
Prednisone, tacrolimus, MMF/MPA as primary immunosuppression regimen [n (%)]	64 (94.1)	9 (90.0)	0.506
Duration of VGCV prophylaxis, days [median (IQR)]	91 (90 – 98.3)	9.15 (81 – 99.5)	0.741
Other complications by post-transplant month 12 [n (%)]			
Delayed graft function[b]	39 (57.4)	5 (50.0)	0.740
Development of NODAT	14 (20.6)	1 (10.0)	0.428
Development of *de novo* DSA	6 (9.0)	1 (10.0)	1.000
Renal artery stenosis requiring revascularization	5 (7.4)	1 (10.0)	0.769
Biopsy-proven acute graft rejection	5 (7.4)	2 (20.0)	0.219

ATG: antithymocyte globulin; BMI: body mass index; CMV: cytomegalovirus; D: donor; DCD: donation after circulatory death; DSA: donor-specific antibody; EBV: Epstein-Barr virus; EBNA: EBV nuclear antigen; HLA: human leukocyte antigen; HCV: hepatitis C virus; IQR: interquartile range; MPA/MMF: mycophenolic acid / mycophenolate mofetil; NODAT: new-onset diabetes mellitus after transplantation; R: recipient; SD: standard deviation; VGCV: valganciclovir.

[a] Bold characters denote significant differences.

[b] Includes coronary artery disease, carotid artery stenosis, abdominal aortic aneurysm, and/or peripheral arterial disease of lower limbs.

Table S5. Clinical factors associated with SDF-1α downregulation upon whole blood stimulation with CMV-specific viral peptides (n = 78).

Variable	SDF-1α downregulation (n = 46)	No SDF-1α downregulation (n = 32)	*P*-value[a]
Age of recipient, years [mean ± SD]	53.1 ± 11.5	53.4 ± 12.1	0.919
Male gender [n (%)]	25 (54.3)	22 (68.8)	0.201
BMI at transplantation, Kg/m^2 [mean ± SD]	24.8 ± 4.7	26.5 ± 7.7	0.250
Pre-transplant comorbidities [n (%)]			
Hypertension	37 (80.4)	27 (84.4)	0.656
Diabetes mellitus	11 (23.9)	9 (28.1)	0.675
Atherothrombotic disease[b]	11 (23.9)	5 (15.6)	0.373
Coronary heart disease	5 (10.9)	2 (6.2)	0.694
Non-ischemic heart disease	6 (13.0)	7 (21.9)	0.303
Chronic pulmonary disease	3 (6.5)	5 (15.6)	0.262
Cerebrovascular disease	4 (8.7)	3 (9.4)	1.000
Previous kidney transplantation [n (%)]	13 (28.9)	10 (31.2)	0.823
Underlying end-stage renal disease [n (%)]			
Diabetic nephropathy	8 (17.4)	7 (21.9)	0.621
Polycystic kidney disease	8 (17.4)	5 (15.6)	0.837
Glomerulonephritis	3 (6.5)	3 (9.4)	0.685
IgA nephropathy	5 (10.9)	0 (0.0)	0.074
Reflux nephropathy	2 (4.3)	3 (9.4)	0.396
D-/R+ CMV serostatus [n (%)]	6 (13.0)	7 (21.9)	0.303
Positive EBV serostatus (anti-EBNA IgG) [n (%)]	43 (93.5)	31 (96.9)	0.503
Positive HCV serostatus [n (%)]	8 (17.4)	6 (18.8)	0.878
Pre-transplant renal replacement therapy [n (%)]	44 (95.7)	31 (96.9)	1.000

Time on dialysis, months [median (IQR)]	35 (13.5 – 63.2)	27.7 (18.1 – 63.4)	0.376
Single kidney transplantation [n (%)]	46 (100.0)	29 (90.6)	0.065
DCD donor [n (%)]	19 (41.3)	15 (46.9)	0.626
Cold ischemia time, hours [mean ± SD]	18.3 ± 6.4	17.2 ± 6.6	0.505
Number of HLA mismatches [median (IQR)]	4 (3 – 5)	4 (3 – 5)	0.227
Total number of ATG doses [median (IQR)]	4.7 (1.5 – 6.6)	5.6 (1.7 – 6.9)	**0.016**
Prednisone, tacrolimus, MMF/MPA as primary immunosuppression regimen [n (%)]	44 (95.7)	29 (90.6)	0.396
Duration of VGCV prophylaxis, days [median (IQR)]	92 (89 – 96.8)	91 (90 – 101.5)	1.000
Other complications by post-transplant month 12 [n (%)]			
Delayed graft function[b]	26 (56.5)	18 (56.2)	0.981
Development of NODAT	12 (26.1)	3 (9.4)	0.144
Development of *de novo* DSA	3 (6.5)	4 (12.9)	0.430
Renal artery stenosis requiring revascularization	5 (10.9)	1 (3.1)	0.207
Biopsy-proven acute graft rejection	4 (8.7)	3 (9.4)	1.000

ATG: antithymocyte globulin; BMI: body mass index; CMV: cytomegalovirus; D: donor; DCD: donation after circulatory death; DSA: donor-specific antibody; EBV: Epstein-Barr virus; EBNA: EBV nuclear antigen; HLA: human leukocyte antigen; HCV: hepatitis C virus; IQR: interquartile range; MPA/MMF: mycophenolic acid / mycophenolate mofetil; NODAT: new-onset diabetes mellitus after transplantation; R: recipient; SD: standard deviation; VGCV: valganciclovir.

[a] Bold characters denote significant differences.

[b] Includes coronary artery disease, carotid artery stenosis, abdominal aortic aneurysm, and/or peripheral arterial disease of lower limbs.
